# Supplementary material for: Loss of Phosphomannose Isomerase Impairs Growth, Perturbs Cell Wall Integrity, and Reduces Virulence of Fusarium oxysporum f. sp. cubense on Banana Plants
Source: J Fungi (Basel). 2023 Apr 16;9(4):478. doi: 10.3390/jof9040478 (PMC10145770; doi:10.3390/jof9040478)
Supplement: Supplementary file 1 [file jof-09-00478-s001.zip › jof-2292140-supplementary.pdf]

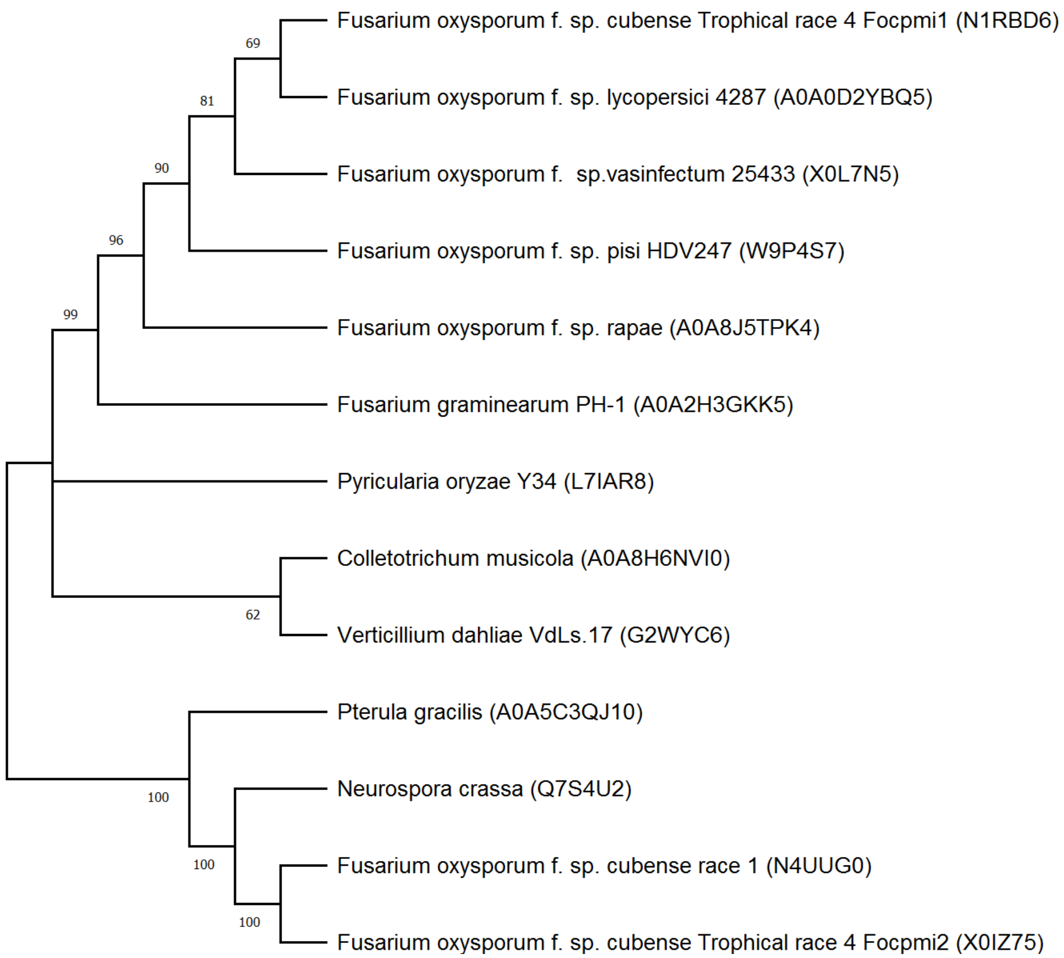

Figure S1. Phylogenetic analysis of PMI proteins among filamentous fungi. PMI Protein sequences from *F. oxysporum* f. sp. cubense TR4 (N1RBD6 and X0IZ75), *F. oxysporum* f. sp. pisi HDV247 (W9P4S7), *F. oxysporum* f. sp. vasinfectum (X0L7N5), *F. oxysporum* f. sp. lycopersici 4287 (A0A0D2YBQ5), *F. oxysporum* f. sp. rapae (A0A8J5TPK4), *F. oxysporum* graminearum PH-1 (A0A2H3GKK5), *Pyricularia oryzae* Y34 (L7IAR8), *Colletotrichum musicola* (A0A8H6NVI0), *Verticillium dahlia* Vdls.17 (G2WYC6), *Pterula gracilis* (A0A5C3QJ10), *F. oxysporum* f. sp. cubense race 1 (N4UUG0) and *Neurospora crassa* (Q7S4U2) sequences were downloaded from NCBI and analyzed by MEGA 11 Neighbor joining method with a bootstrap value of 5,000 replicates.

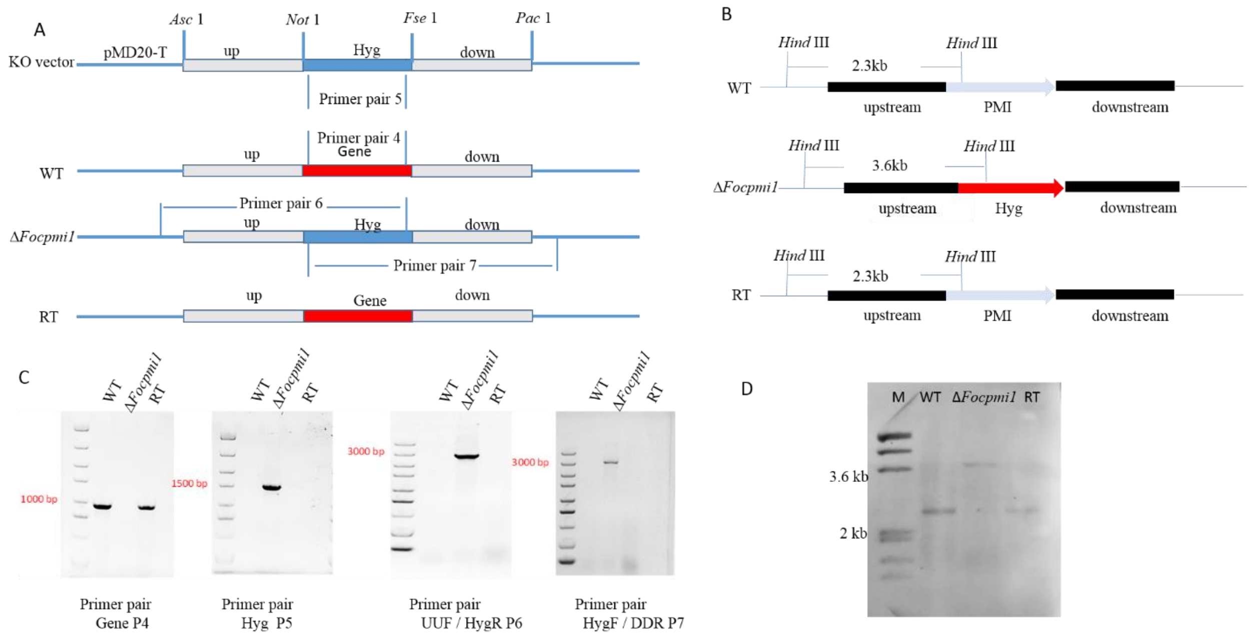

Figure S2. Schematic representation of strategies for the production and confirmation of Δ*Focpmi1* and RT strains. A, Strategies to generate the *ΔFocpmi1* mutant and revertant (RT) strains by homologous recombination. WT is wild-type, *ΔFocpmi1* is the mutant, and RT is the revertant strain. B, Genomic cutting sites for southern blotting. C, PCR analysis using four pairs of primers indicated in A. D, verification of the Δ*Focpmi1* mutant and RT strains by southern blotting.

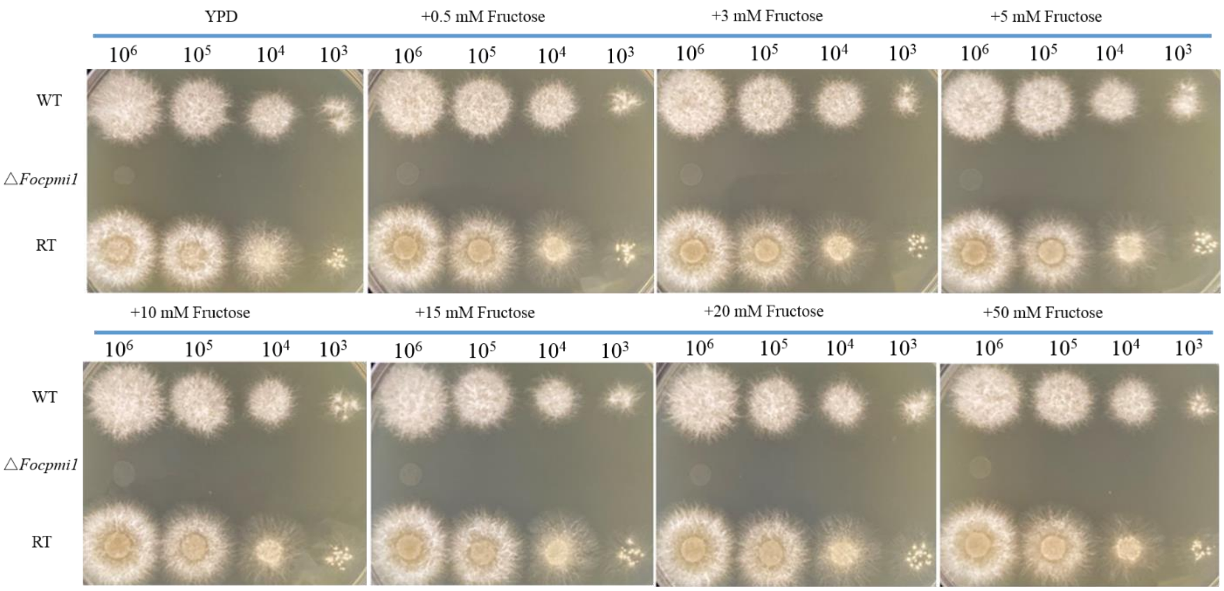

Figure S3. Growth characterization of the Δ*Focpmi1* at different concentrations of fructose. Freshly harvested conidia of all the three strains were serially diluted (10^6-10^3) and point inoculated on YPD medium amended with different concentrations (0.5, 3, 5, 10, 15, 20 and 50mM) of fructose. The plates were incubated at 28 °C for 48 hours.

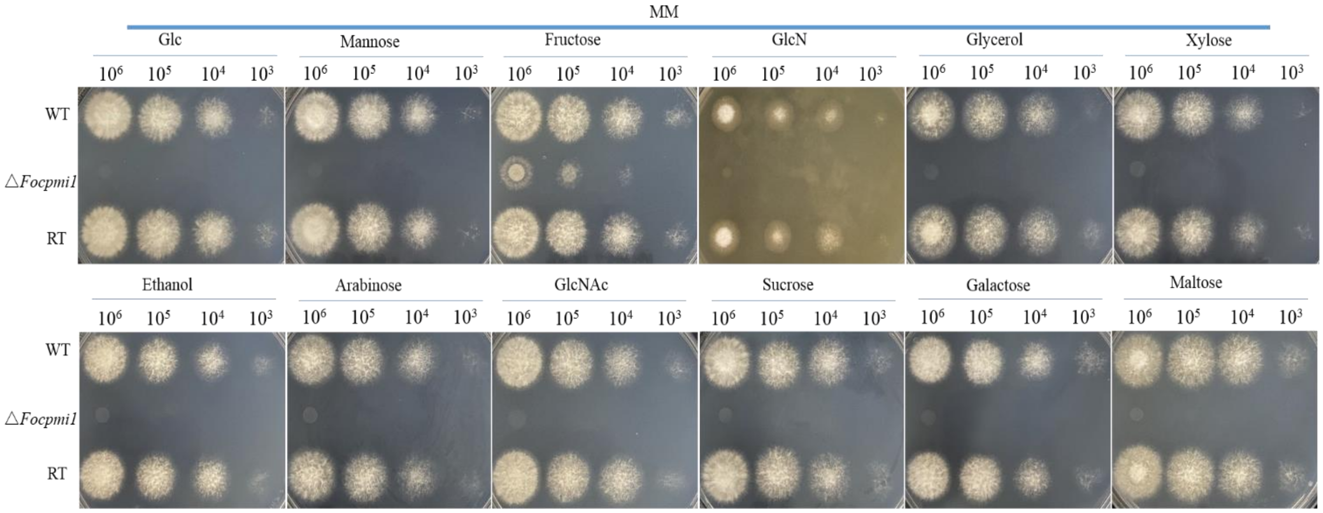

Figure S4. Growth characterization of the *Focpmi1* mutant on different sugars as sole carbon sources. Freshly harvested conidia from all the three strains were point inoculated on MM solid medium plates containing Glc, mannose, fructose, GlcN, glycerol, xylose, ethanol, arabinose, N-acetylglucosamine (GlcNAc), Sucrose, galactose and maltose. The plates were incubated at 28 °C for 48 hours.

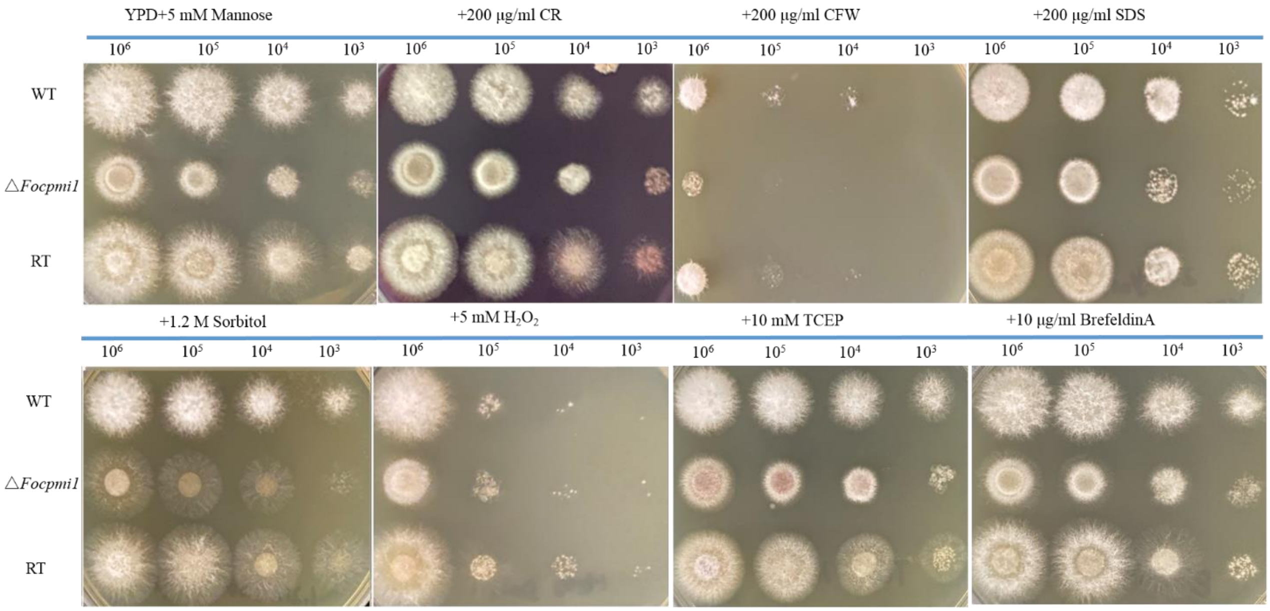

Figure S5. Cell wall integrity analysis and sensitivity towards osmotic, and ER stress in YPD medium. Freshly harvested serially diluted conidia 10^6-10^3 were point inoculated onto YPDM plates supplemented with 300 μg/ml CR, 50 μg/ml CFW, 200 μg/ml SDS, 1.2 M sorbitol, 5 mM H_2O_2, 10 mM TCEP and 10 μg/ml Brefeldin A. The plates were photographed after 48 hours of incubation at 28 °C.

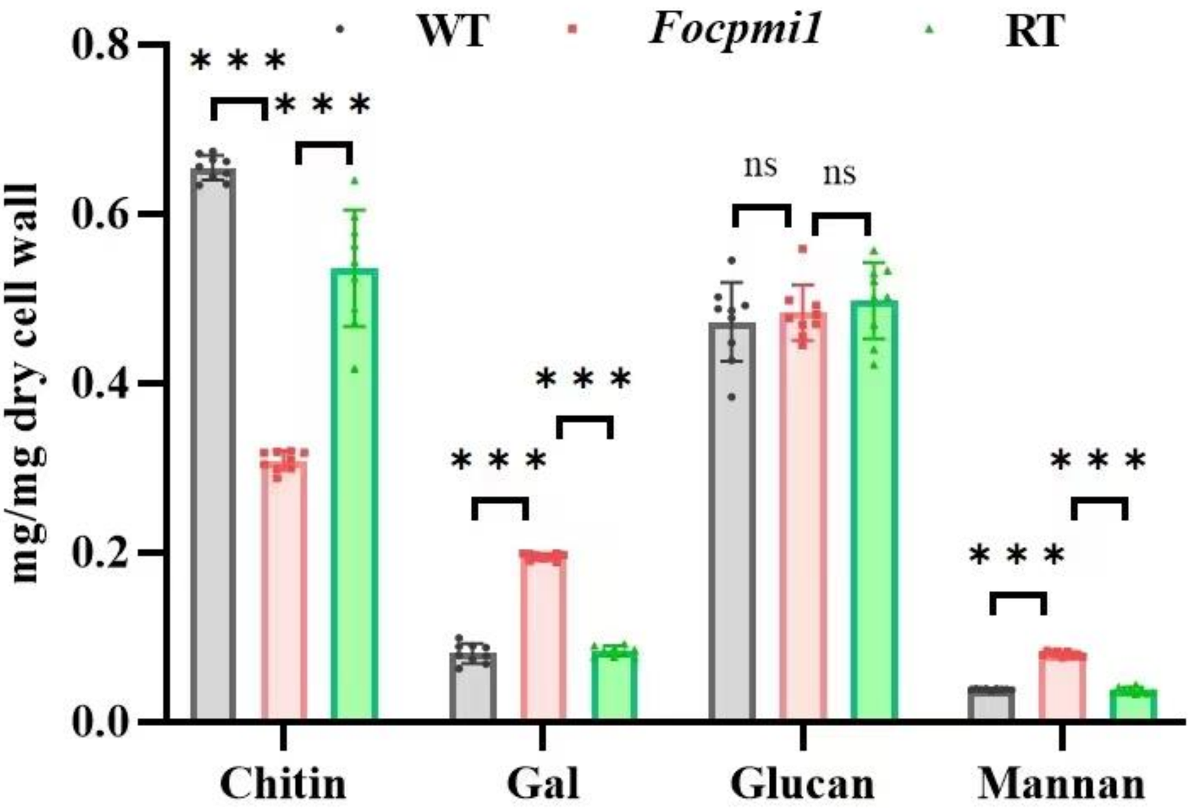

Figure S6. Cell wall content analysis of Δ*Focpmi1* strain. 2×10^{8} conidia of WT, Δ*Focpmi1* and RT strains were inoculated in MMM liquid medium and incubated at 28 °C for 72 hours. Dried mycelia (10 mg) were used for the cell wall contents quantification. The experiment was conducted in three biological replicates.

Table S1. Primers used in the study

Primers	Primer sequences (5/-3/)	Length (bp)	Remark
**P1**			
Focpmi1-up-F (*Asc*I)	TT GGCGCGCC GAA GCA CAT CAA GCG TTG GTT	1185	*Focpmi1* mutant construction
Focpmi1-up-R (*Not*I)	ATAAGAATGCGGCCGCATTTGCGATGTTCGTTGTTGG		
**P2**			
Hyg-F (*Not*I)	ATAAGAATGCGGCCGCGACAGAAGATGATATTGAAGG	1429	*Focpmi1*mutant construction
Hyg- R (*Fse*I)	TATGGCCGGCCGATTTCAGTAACGTTAAGTGG		
**P3**			
Focpmi1-down-F (*Fse*I)	TAT GGCCGGCC ATGATATTGGAGTGATGTGAG	1119	*Focpmi1*mutant construction
Focpmi1-down-R (*Pac*I)	CC TTAATTAA CGA CGT AAT ATC TCG ACT ATG		
**P4**			Mutant and revertant confirmation
Focpmi1 Gene-F	TCTCCTCCCACTACGGCAACA	944	
Focpmi1 Gene-R	AGCGATCCTTCCCTTTCCACT		
**P5**			Mutant and revertant confirmation
Hyg-F	GACAGA AGATGATATTGAAGG	1429	
Hyg-R	GATTTCAGTAAC GTTAAGTGG		
**P6**			Mutant and revertant
Focpmi1-UUF	GGGCATTCCACTGTCTGTTGT	2865	

Hyg-R	GAT TTC AGT AAC GTT AAG TGG		confirmation
**P7**<br>Hyg-F<br>*Focpmi1*-DDR	GAC AGA AGA TGA TAT TGA AGG<br>CCTGTCCGACATAGCTGTTCCA	2874	Mutant and revertant confirmation
**P8**<br>*Focpmi1*-UUF<br>*Focpmi1*-DDR	GGGCATTCCACTGTCTGTTGT<br>CCTGTCCGACATAGCTGTTCCA	4286	Revertant construction
**P9**<br>*Focpmi1* RT-F<br>*Focpmi1* RT-R	AAGGTGTTACGGATGAGAAGAAGAAGT<br>GAAGAAGAGCACAAAGCAACCAAT	236	qRT-PCR
**P10**<br>*Focpmi2* RT-F<br>*Focpmi2* RT-R	TACGCCAGAAGCCTTTGAGAC<br>TAGGTTGCCAGGGTCGTTTGC	246	qRT-PCR
**P11**<br>*FocEF1α*- F<br>*FocEF1α* -R	CCCAAGGCTGTCGCTTTCGTC<br>AGACGGAGGGGCTTGTCAACG	191	qRT-PCR
**P12**<br>*Focpmi2*-up-F (*Asc*I)	TT GGCGCGCC GACATTTGCAATACTTAGACG	1147	*Focpmi2* mutant construction

Focpmi2-up-R (*Not*I)	ATAAGAATGCGGCCGCATGACTATGACGATTAAC GAA		
**P13**<br>*Focpmi2*-down-F (*Fse*I)<br>*Focpmi2*-down-R (*Pac*I)	TAT GGCCGGCC CGA CAT TTT GAG ATT CAA TGC<br>CC TTAATTAA ATC GAC ATC CTG AGT TTA TAG	1190	*Focpmi2* mutant construction

Table S2. Genes responsible for plant cell wall degrading enzymes.

Gene ID	FDR	log2FC	Predicted function
Focb16_v004426	2.10E-06	-7.2308	Carboxylic ester hydrolase
Focb16_v000085	2.38E-11	-2.44834	Carboxylic ester hydrolase
Focb16_v011188	1.22E-06	-7.33859	Acetylxylan esterase 2
Focb16_v014173	3.95E-05	-2.41913	Alpha-glucosidase 2
Focb16_v000244	0.000414	-3.42482	Beta-glucosidase 4
Focb16_v000177	6.41E-12	-2.80639	Glycerophosphodiester phosphodiesterase
Focb16_v003281	1.22E-16	-3.98028	Beta-glucosidase
Focb16_v011585	9.11E-09	-2.50828	Putative beta-glucosidase C
Focb16_v002629	5.63E-16	-2.5294	Mannan endo-1,6-alpha-mannosidase
Focb16_v014263	3.40E-10	-5.97382	Beta-mannosidase B

Focb16_v015639	1.19E-35	-5.91848	Mannan endo-1,6-alpha-mannosidase
Focb16_v002729	1.77E-23	-6.59788	Endo-beta-1,4-glucanase D
Focb16_v004733	2.41E-08	-4.95047	Putative endo-beta-1,4-glucanase D
Focb16_v007553	9.92E-24	-4.15232	Endoglucanase-4
Focb16_v009544	7.22E-23	-2.35588	chitinase
Focb16_v005330	1.06E-15	-6.31701	Beta-L-arabinobiosidase
Focb16_v004994	0.002243	-5.13582	alpha-L-fucosidase
Focb16_v011413	0.000117	-4.32552	Alpha-L-fucosidase 2
Focb16_v000310	1.08E-56	-4.63723	Putative pectin lyase D
Focb16_v003939	2.07E-63	-3.33653	Glycosidase
Focb16_v008003	2.80E-05	-3.76292	Peptidoglycan deacetylase

Focb16_v011761	3.17E-37	-4.11241	Glycosidase
Focb16_v012948	7.03E-07	-7.4379	Unsaturated glucuronyl hydrolase
Focb16_v013479	2.70E-45	-2.96048	Phosphotransferase
Focb16_v013482	2.16E-17	-4.11972	N-acetylglucosamine-6-phosphate deacetylase
